# When and how to intervene to improve the health of children born HIV‐free

**DOI:** 10.1002/jia2.26157

**Published:** 2023-11-01

**Authors:** Ceri Evans, Andrew J. Prendergast

**Affiliations:** ^1^ Zvitambo Institute for Maternal and Child Health Research Harare Zimbabwe; ^2^ Institute of Infection Veterinary and Ecological Sciences University of Liverpool Liverpool UK; ^3^ Blizard Institute Queen Mary University of London London UK

1

Children born to mothers with HIV, who themselves remain HIV‐free, are at risk for poorer health outcomes compared to children born to mothers without HIV, including increased morbidity and mortality, and impaired growth and neurodevelopment [1]. However, there is limited evidence to inform interventions for this population (termed children born HIV‐free).

Here, we highlight intervention options targeting either HIV‐specific or universal risk factors, which may be impactful when delivered to the mother or infant (Figure 1).

**Figure 1:**
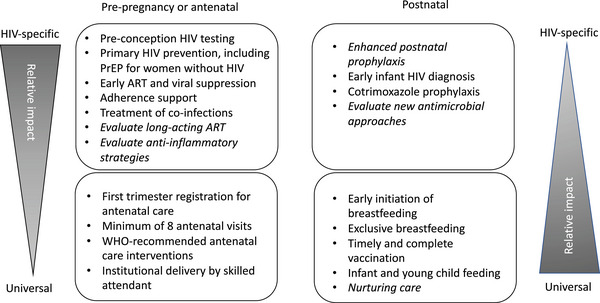
When and how to intervene. HIV‐specific and universal approaches to improve the clinical outcomes of children born HIV‐free are shown according to the timing of intervention in the life cycle. The interventions shown in italics are plausible approaches to improve outcomes but require further evaluation. The thickness of the wedge shows the likely relative impact of each intervention. Abbreviations: ART, antiretroviral therapy; PrEP, pre‐exposure prophylaxis.

Since advanced maternal HIV is associated with poor outcomes among children born HIV‐free [1], maternal HIV‐specific interventions should be started as soon as possible. Women of childbearing age should be able to access pre‐conception HIV testing. Those without HIV in high‐burden settings, and those in low‐burden settings with a high risk of HIV acquisition, should be offered pre‐exposure prophylaxis and other primary HIV prevention strategies, including repeat HIV testing, while ensuring that women living with HIV are retained in care and accessing antiretroviral therapy (ART). Where viral load testing is available during pregnancy, detectable viraemia should prompt urgent interventions to rapidly reduce HIV to undetectable levels. This is essential to prevent vertical HIV transmission, but may also be important for children born HIV‐free, since antenatal HIV viraemia has been correlated with higher risk of child mortality [2] and suboptimal neurodevelopment [3]. Newer antiretrovirals, including injectable long‐acting agents, may optimise adherence, but these agents need to be evaluated for use during pregnancy [4], with nested evaluation of short‐ and long‐term child health outcomes.

Maternal systemic inflammation during pregnancy has been associated with infant mortality in a pathway independent of HIV viraemia [5], suggesting that interventions beyond ART might reduce infant mortality. Two approaches could be considered: evaluating anti‐inflammatory agents, or addressing the underlying drivers of inflammation, including sub‐clinical co‐infections. Maternal co‐infections may themselves drive adverse outcomes in children born HIV‐free. For example, cytomegalovirus (CMV) viraemia during pregnancy has been associated with infant mortality, and with earlier CMV acquisition and immune activation among surviving infants [5]. Antenatal valacyclovir is, therefore, a logical strategy, but did not delay infant cytomegalovirus acquisition in HIV‐affected mother‐infant pairs in Kenya [6]. Newer, less toxic agents, such as letermovir, warrant evaluation during pregnancy, with infant mortality as the primary outcome, and infant CMV acquisition and immune activation as secondary outcomes.

First‐trimester registration for antenatal care, a minimum of eight antenatal contacts and institutional delivery by a skilled attendant are recommended to reduce maternal deaths, newborn mortality and stillbirths [7], but priority interventions have low coverage across low‐ and middle‐income countries (LMICs) [8]. For example, the median coverage of at least four antenatal visits in LMICs is below 80% [8]. Other evidence‐based interventions [7] should focus on reducing the proportion of infants born preterm and small‐for‐gestational age, since these outcomes are more common among women living with HIV and are associated with increased infant morbidity, mortality and neurodevelopmental delays [1].

Current interventions for infants include postnatal antiretroviral prophylaxis to reduce HIV transmission, and co‐trimoxazole (trimethoprim‐sulfamethoxazole), which reduces mortality in children who acquire HIV; however, neither intervention has specific benefits for children born HIV‐free. Two trials from non‐malarial areas in sub‐Saharan Africa show that co‐trimoxazole did not reduce mortality among children born HIV‐free [9], and some countries with effective programmes for the prevention of vertical transmission and early infant diagnosis may, therefore, stop providing universal co‐trimoxazole. However, there are emerging data that excess mortality among children born HIV‐free occurs in the neonatal period [10], so future trials could explore starting co‐trimoxazole from birth. The safety concern regarding sulfamethoxazole‐induced kernicterus is theoretical [11], and co‐trimoxazole was safe when given to children from 2 weeks of age in Botswana [9]. Azithromycin is an alternative broad‐spectrum antibiotic, although a large multi‐country randomised‐controlled trial of providing single‐dose azithromycin to women in labour did not reduce neonatal sepsis [12]; however, women living with HIV were not specifically evaluated and infants did not receive azithromycin. In the MORDOR trial, azithromycin reduced infant deaths in high‐mortality settings [13]. Clearly, antimicrobial stewardship concerns would need to be considered to avoid the emergence of resistance, especially in countries where a substantial proportion of children are born to women living with HIV.

Overcoming the additional risks and vulnerabilities among families affected by HIV is critical to ensure that children born HIV‐free reach their full potential. Nurturing care comprises five inter‐related elements to protect children from adversity and ensure they can thrive: good health, adequate nutrition, safety and security, responsive caregiving and early learning. Optimal breastfeeding helps all children survive and thrive, including children born to women living with HIV in sub‐Saharan Africa. A South African study demonstrated reduced hospitalisation among children born HIV‐free whose mothers initiated early and exclusive breastfeeding [14]; in the same study, timely and complete childhood immunisation was also associated with fewer hospitalisations [14]. However, in surveys across 36 LMICs, the median coverage of exclusive breastfeeding from birth through 5 months was below 50%, and coverage of complete vaccination series for diphtheria, tetanus, pertussis, measles and polio was below 80% [12]. Children born HIV‐free have a high risk of stunting [1], which can be reduced with improved infant and young child feeding, including small‐quantity lipid‐based nutrient supplements [15]. These supplements may improve child survival, stunting, wasting and neurodevelopment, so it is particularly important that the global impetus to scale‐up their use includes areas with high antenatal HIV prevalence, as children born HIV‐free may particularly benefit [15]. Additional universal interventions include addressing parental mental health, family planning, prevention and cessation of smoking, alcohol and other substance use; and child deworming, healthy hygiene practices, neurodevelopmental screening and play.

Improving the outcomes of children born HIV‐free requires identifying those most at risk and providing a comprehensive package of HIV‐specific and universal interventions. Current HIV‐specific interventions appear most effective when delivered during pregnancy, while available postnatal strategies principally address universal risk factors (Figure 1). New strategies addressing the underlying HIV‐specific and universal risks are needed, which can be integrated with existing interventions to create a wraparound nurturing care package delivered through existing contacts with health services to help children born HIV‐free to reach their full potential.

## COMPETING INTERESTS

The authors have no competing interests to declare.

## AUTHORS’ CONTRIBUTIONS

AJP wrote the first draft of the manuscript. CE designed the figure and critically revised the manuscript. Both authors have read and approved the final manuscript.

## Data Availability

Data sharing not applicable to this article as no datasets were generated or analysed during the current study.
